# The Immunomodulatory Imbalance in Patients with Ketamine Cystitis

**DOI:** 10.1155/2017/2329868

**Published:** 2017-10-24

**Authors:** Gang-Yi Fan, Juin-Hong Cherng, Shu-Jen Chang, Raju Poongodi, Adrienne Chang, Sheng-Tang Wu, En Meng

**Affiliations:** ^1^Graduate Institute of Medical Sciences, National Defense Medical Center, Taipei, Taiwan; ^2^Department of Biology and Anatomy, National Defense Medical Center, Taipei, Taiwan; ^3^Faculty of Dentistry, School of Dentistry, National Yang-Ming University, Taipei, Taiwan; ^4^Department of Chemistry, New York University Abu Dhabi, Abu Dhabi, UAE; ^5^Division of Urology, Department of Surgery, Tri-Service General Hospital, National Defense Medical Center, Taipei, Taiwan

## Abstract

The pathogenesis of ketamine cystitis (KC) has been recently linked with immune response to patients but the same has not yet been established. Hence, this study aims to propose a possible immune mechanism of irreversible bladder damage caused by KC. A total of 53 KC patients and 21 healthy volunteers as controls have been retrospectively assessed. The levels of serum immunoglobulin E (IgE), IL-6, and IFN-*γ* of KC patients were significantly higher than those of controls, whereas the TGF-*β* levels of KC patients substantially reduced but the IL-2 and IL-4 levels of KC patients were comparable to those of controls. Moreover, the KC patients had significantly higher counts of T_H_1, T_H_2, and T_H_17 cells than those of controls. The immune response of KC users may begin with the IL-6 production and differentiation of T_H_17 and may be followed by alternating between high expressions of T_H_1 and T_H_2. The IL-6 may further suppress the T_REG_ cells which can aggravate chronic inflammation in KC patients and the imbalance in T_H_17 and T_REG_ cells may involve the pathogenesis of KC. Further investigation is needed to define the role of IL-6 in T_H_1/T_H_2/T_H_17-regulated signaling pathway in ketamine-induced cystitis.

## 1. Introduction

Ketamine is a phencyclidine hydrochloride derivative which is mainly used for starting and maintaining anesthesia [1]. It induces a dissociative, trance-like state in which the patient is unable to respond to external stimuli [2]. Chronic abuse has been associated with an out-of-body experience known as the K-hole phenomenon [3]. Due to low price and availability, ketamine has been increasingly used as a recreational drug which affects the central nervous and cardiovascular systems [4]. The chronic ketamine abuse may cause ulcerative cystitis and dysfunction of the lower urinary tract, both of which have been recognized as a new disease entity, called “ketamine cystitis” (KC). The number of KC patients has been increased dramatically in the past decade [5]. The KC shares many common histopathological features with interstitial cystitis/bladder pain syndrome (IC/BPS), including urothelial ulceration, inflammatory cell infiltration, and varying degrees of bladder wall fibrosis [6]. Among them, the long-term bladder inflammation may cause fibrosis of the bladder wall and, eventually, a contracted bladder [7]. In particular, the eosinophil and mast cell infiltration are frequently seen in human bladder tissue after long-term ketamine use [6, 8, 9]. A recent study reported that the KC patients had higher serum immunoglobulin E (IgE) than patients with IC/BPS or acute bacterial cystitis, which may be associated with bladder pain severity and small maximal bladder capacity in KC patients [10]. Therefore, the immune response to ketamine may play a key role in the pathogenesis of KC. However, only little knowledge on the immune activity in KC patients has been established.

On the other hand, the pivotal immune events of immune diseases are the development of antigen-specific effector T helper type 2 (T_H_2) cells, T_H_1 cells, or the recently defined T_H_17 cells that are associated with clinical features and disease progression [11]. It is known that the phenotype of T cells is influenced by the tissue microenvironment which is produced through the dominant kind of cytokines, costimulatory molecules, and the character and dose of antigen presented. The T_H_1 cell development is promoted by interleukin 12 (IL-12) and gamma interferon (IFN-*γ*) and primarily involves T-bet signaling. The generation of T_H_2 cells is probably induced by IL-4, IL-5, and IL-6 costimulation. The T_H_17 lineage is induced by the secretion of IL-6, IL-21, and the transforming growth factor beta (TGF-*β*), whereas the T_H_17 cells secrete a profile of potent proinflammatory cytokines (IL-17, IL-21, and IL-22), potent tumor necrosis factor *α* (TNF-*α*), and IL-6 upon certain stimulation [12]. It is to be noted that the regulatory T (T_REG_) cells secrete IL-10 and TGF-*β*, which modulates helper T cell activity and suppresses some of their functions, inducing tolerance to antigens.

The T_H_1, T_H_2, and T_H_17 populations, and the cytokines they release, are antagonistic to each other and one or the other subtype is dominant in response to a particular pathogen at any one time. However, it is important to know how the body determines, and which differentiation pattern and immune response are appropriate for a specific pathogen, and what the molecular mechanisms that underpin differentiation into a T_H_1, a T_H_2, or a T_H_17 cell are [13]. Since the contribution of specific T_H_ cell subsets and related cytokines in the pathogenesis of KC is unknown, we hypothesize that measuring circulating cytokines may elucidate the imbalance between T_H_ cell subsets in KC patients. In this context, we conducted this case-control study in order to investigate plasma levels of T_H_1 (IL-2, IFN-*γ*), T_H_2 (IL-4, IL-5), T_H_17 (IL-17), and T_REG_ (TGF-*β*) cytokines.

## 2. Materials and Methods

### 2.1. Patient and Sample Collection

A total of 53 KC patients and 21 healthy volunteers were included in this retrospective study. Each of the KC patients showed symptoms of lower urinary tract irritation, such as urgency, nocturia, and frequency. The urine and blood samples of each patient were collected for analysis. The urine test panel was conducted for pH and sediments which include urine red blood cells (RBCs), urine white blood cells (WBCs), and epithelial cells. Blood analysis included WBC counts, RBC counts, and differential count (neutrophils, lymphocytes, monocytes, and eosinophils). The paraffin-embedded urothelial tissues were obtained by urothelial biopsy from six patients under sterile conditions after one week of the urine and blood sample collection.

### 2.2. Histological Analysis

The paraffinized sections (0.6 *μ*m) of each patient (3–10 sections/patient) were stained with Gill's hematoxylin V (MUTO, Tokyo, Japan) and 1% eosin alcohol solution (MUTO; H&E). The obtained H&E slides were examined under a light microscope (Olympus BX51; Olympus, Tokyo, Japan).

### 2.3. Serum Cytokine Assay

The serum samples were collected and the production of cytokine/chemokines was quantified by the MILLIPLEX™ MAP Human Cytokine/Chemokine Kit (Millipore Corp., Billerica, MA). Serum specimens were drawn from 21 KC patients (13 males, 8 females) and 21 volunteers (17 males, 4 females). Eight-parameter logistic standard curves were set and data were processed with MILLIPLEX® Analyst 5.1 software. The cytokines levels IL-1, IL-2, IL-4, IL-5, IL-6, IL-17, TNF-*α*, IFN-*γ*, TGF-*β*1, TGF-*β*2, and TGF-*β*3 were analyzed by enzyme-linked immunosorbent assay (ELISA).

### 2.4. T_H_1/T_H_2/T_H_17 Determination in Peripheral Blood Mononuclear Cells (PBMCs)

Peripheral blood was collected from 13 KC patients (10 males, 3 females) and 13 volunteers (10 males, 3 females) by venous puncture and collected into preservative-free heparin. The PBMCs were isolated by means of Ficoll-Plaque Plus density gradient centrifugation (Amersham Biosciences, NJ, USA). For intracellular staining, PMBCs at a concentration of 1–10 million cells/mL were stained for 5 h with phorbol 12-myristate 13-acetate (PMA)/ionomycin at 50 ng/mL and 1 *μ*g/mL, respectively. 4 *μ*L of BD GolgiStop™ was added for every 6 mL of cell culture and mixed thoroughly. The PBMCs were then fixed in BD Cytofix™ Fixation Buffer and made permeable with BD Phosflow™ Perm/Wash Buffer. The percentages of T_H_1 and T_H_2 in CD4^+^ T cells were obtained through flow-cytometric analysis using the BD Human T_H_1/T_H_2/T_H_17 Phenotyping Kit, which has a four-color cocktail of fluorescent antibodies specific for Human CD4 (PERCP-CY5.5 Clone: SK3), IFN-*γ* (for T_H_1, FITC Clone: B27), IL-4 (for T_H_2, APC Clone: MP4-25D2), and IL-17A (for T_H_17, PE Clone: N49-653).

### 2.5. Statistical Analysis

All the data were evaluated by analysis of variance (Sigma plot, 2001). The values are reported as mean ± SD of at least three experiments. Paired *t*-test and chi-square test were employed for comparison between two groups, and *p* values ≤ 0.05 were considered statistically significant. The correlation coefficient (*r*) was applied between the serum cytokine levels.

## 3. Results

**Figure 1:**
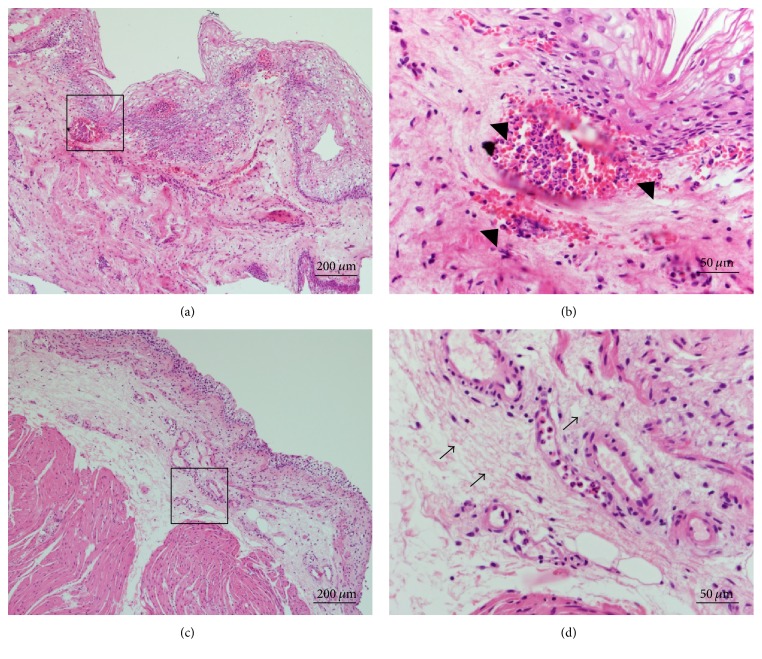
Histological analysis of urothelial bladder biopsy in ketamine cystitis patients stained with hematoxylin and eosin demonstrating. (a, b) Neutrophils and eosinophils infiltrates in suburothelium and (c, d) fibrosis deposition (magnification, 100x). The stroma of urothelial bladder biopsy (b) and (d) designated by the black boxed section in (a) and (c), respectively (magnification, 400x). The arrow denotes “neutrophils and eosinophils infiltrates” and “fibrosis deposition” in Figure 1(b) and Figure 1(d), respectively.

A total of 74 subjects (53 KC users and 21 nonusers) were enrolled in this study. The chronic ketamine users are 31 males and 22 females in which their age was 23.84 ± 4.74 years (mean ± SD). The average duration of ketamine usage was 50.44 ± 4.99 months for KC patients (Table 1). Among the 53 KC patients, 26 patients underwent full clinical examination and cystoscopy with hydrodistention, and 3 patients refused bladder biopsy.  Table 2 lists the clinical information and serum Ig levels. The laboratory studies revealed an elevated creatinine (1.25 ± 0.42 mg/dL) and IgE levels (261.59 ± 56.03 IU/mL) with positive RBCs and WBCs in urine analysis. Further, the urothelial biopsies were performed a week after urine and blood sample collection. And, the tissue histology analyzed using hematoxylin and eosin (H&E) stain. The urinary bladder mucosa revealed denuded bladder epithelium, hemorrhage (Figures 1(a) and 1(c)), acute and chronic inflammation as well as the presence of eosinophil, eosinophil infiltrates, and fibrosis deposition over submucosa; the stroma revealed intravascular neutrophil and eosinophil accumulation (magnified view from black box, Figures 1(b) and 1(d)).

**Figure 2:**
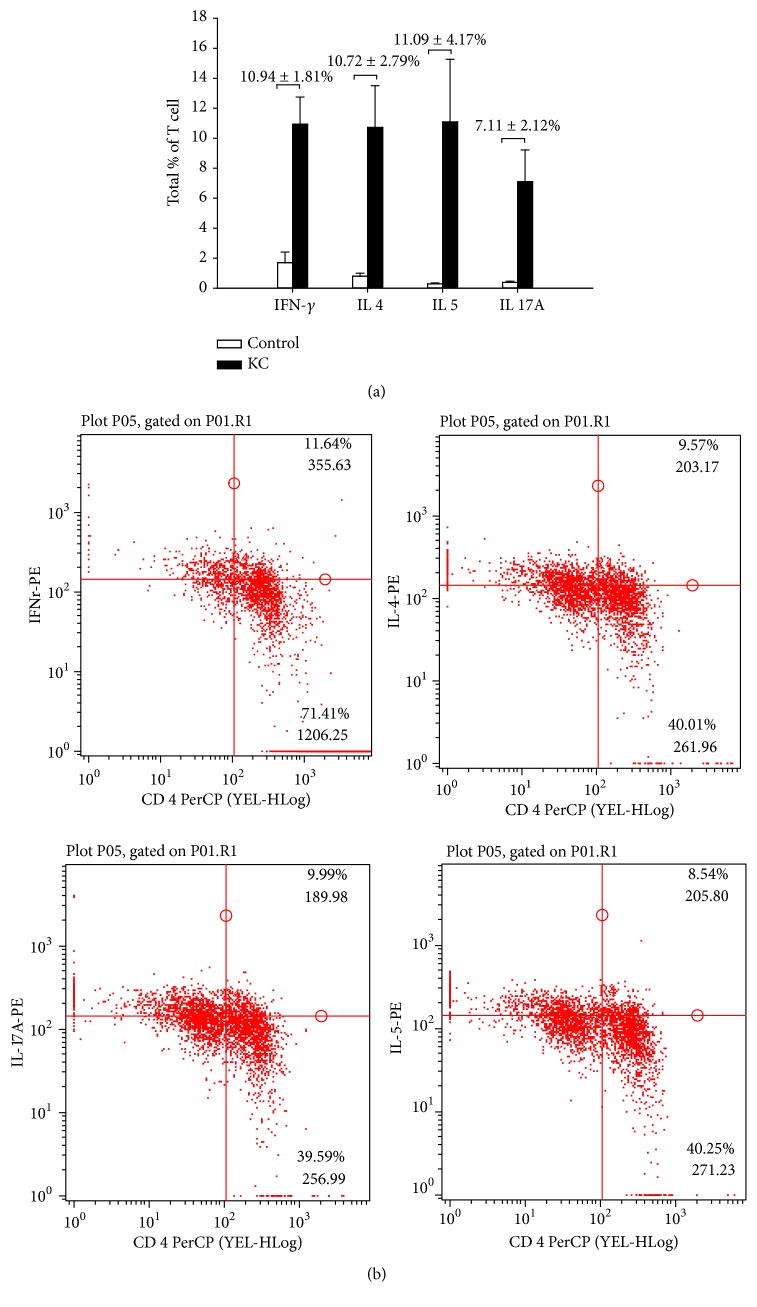
Elevated CD4^+^T_H_1 (IFN-*γ*), T_H_2 (IL-4 and IL-5), and T_H_17 (IL-17a) populations in human PBMC of KC patients versus healthy volunteers. Peripheral T cells were stimulated with 1 ng/ml PMA, 1 *μ*M ionomycin, and 3 *μ*M monensin. Intracellular staining (FACS) was done after 6 hours. Some representative FACS plots are given in (b).

**Figure 3:**
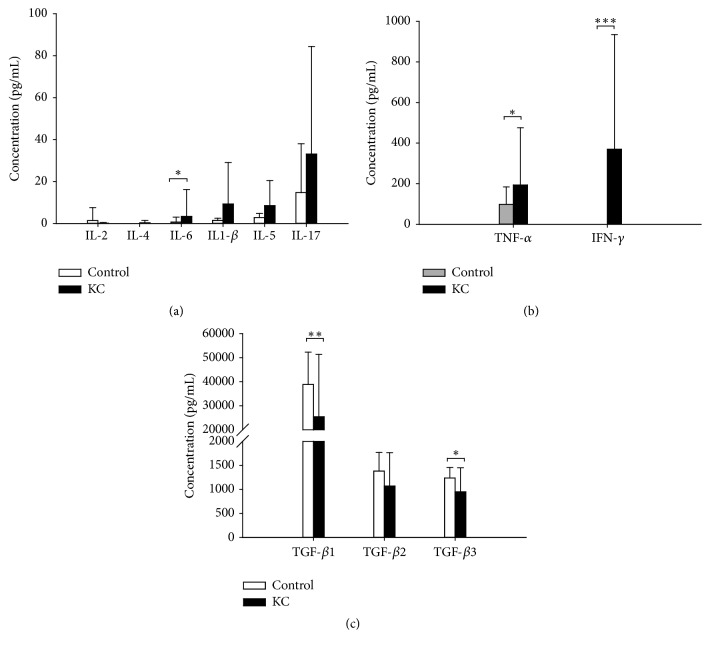
Effects of ketamine on inflammatory cytokines production by serum cytokine analysis. Data are expressed as mean ± SD, where *n* = 27. KC patient shows significantly higher concentrations of cytokines IL-6 (a), TNF-*α*, IFN-*γ* (b) and significantly lower concentrations of TGF-*β*1 and TGF-*β*3 (c). ^*∗*^*P* < 0.05, ^*∗∗*^*P* < 0.01, and ^*∗∗∗*^*P* < 0.001.

Flow-cytometric analysis of KC patients showed considerably higher T_H_1 (IFN-*γ*, 10.94 ± 1.81%), T_H_2 (IL-4, 10.72 ± 2.79% , and  IL-5, 11.09 ± 4.17%), and T_H_17 (IL-17A, 7.11 ± 2.12%; *p* = 0.0001) cells and also gave some representative fluorescence-activated cell sorting (FACS) plots (Figure 2). The univariate comparison of 6 cytokines in the normal and KC groups revealed a two- to ninefold increase in serum IL-6 (2-tailed *p* = 0.05), IL-1*β*, IL-5, and IL-17 expression. The serum levels of the remaining proteins, namely, IL-2 and IL-4, were not significantly increased in the KC group relative to control groups (Figure 3(a)). The distribution of all serum cytokines tested passed the Mann–Whitney *U* test for normality. In addition, the serum cytokine assays were used to determine the effects of ketamine on inflammatory cytokine levels which showed a remarkably increased level of TNF-*α* (KC versus control; 193.59 ± 282.17 versus 98.43 ± 85.48 pg/mL) and IFN-*γ* (369.78 ± 564.56 versus 0 pg/mL; *p* < 0.001) in KC cases (Figure 3(b)).

Patients with KC had significant decrease in TGF-*β*1 (KC versus control; = 25429.14 ± 25940.15 versus 38857.60 ± 13482.19 pg/mL; *p* = 0.008) and TGF-*β*3 (950.01 ± 498.500 versus 1236.30 ± 218.88 pg/mL; *p* = 0.046; Mann–Whitney *U* test) levels (Figure 3(c)). The TGF-*β*2 level was also found to lower in KC patients comparing to the controls (1068.22 ± 695.280 versus 1380.99 ± 387.45 pg/mL; *p* = 0.093) although there was no significance. Since the KC group revealed that the levels of TGF-*β*1, TGF-*β*2, TGF-*β*3 were below the level of detection in the control group, these results lend support to our hypothesis that an increase in cytokines is linked to inflammatory cells and regulation between T_H_1, T_H_2, and T_H_17.

**Figure 4:**
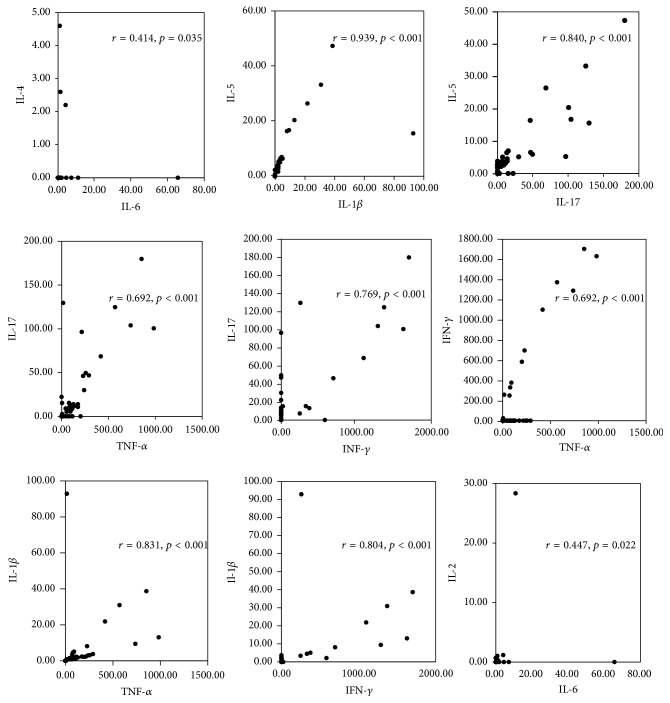
Correlation between various circulating cytokines in patients with ketamine cystitis.

The basis of several statistical tests that result in a correlation coefficient is defined as a numerical representation of the strength and direction of a relationship. On moving to the correlation of cytokines of KC patients (Figure 4), the IL-6 was positively correlated with IL-4 (*r* = 0.414, *p* < 0.05), and IL-1*β* and the IL-17 were positively correlated with IL-5 (*r* = 0.939, *p* < 0.001 and *r* = 0.840, *p* < 0.001). Further, the TNF-*α* and IFN-*γ* were positively correlated with IL-1*β* (*r* = 0.831, *p* < 0.001 and *r* = 0.804, *p* < 0.001), and TNF-*α* and IFN-*γ* were positively correlated with IL-17 (*r* = 0.692, *p* < 0.001 and *r* = 0.769, *p* < 0.001). Moreover, a direct correlation was found in TNF-*α* with IFN-*γ* (*r* = 0.692, *p* < 0.001) and IL-6 with IL-2 (*r* = 0.447, *p* < 0.01). But, IL-6 with IL-4 and IL-6 with IL-2 have found very least correlation in this study.

## 4. Discussion

**Figure 5:**
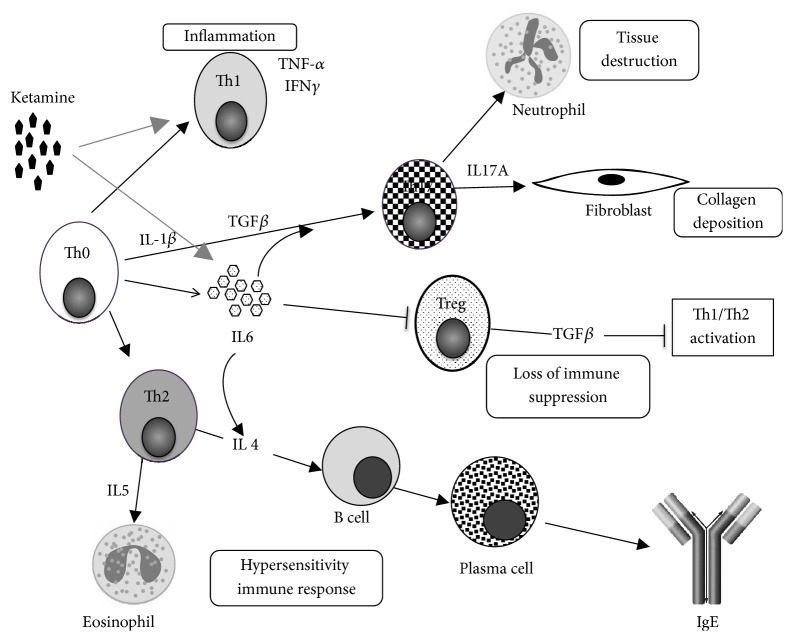
Schematic representation for the relationship between T cell pathways and their effector cytokines in ketamine cystitis patients. Ketamine may cause acute inflammation through T_H_1 pathway. Ketamine may also promote IL-6 generation which links to the generation or maintenance of T_H_17 cell effector functions and then causes tissue destruction and collagen deposition. IL-6 promotes the differentiation of T_H_2 cells by enhancing endogenous IL-4 production and increases the production of IgE. IL-6 may also suppress T_REG_ cells and therefore sustain the activation of T_H_1 and T_H_2 cells. Reinforced feedback of IL-6 increases the production of IL-1*β* and IL-17 from CD4+ T cells.

In the current study, we have illustrated the different profiles of inflammatory cytokines and T_H_ cell expression in KC patients versus healthy controls. The differing cytokine characteristics between normal control and chronic ketamine users may allude to a possible mechanism of KC (Figure 5). In this regard, we hypothesize that the acute inflammation seen in the bladder mucosal and submucosal layer may be caused by the acute immune response of ketamine through the T_H_1 cell pathway. Ketamine may enhance the production of IL-6 which can further result in the apoptosis and shedding of epithelial and interstitial cells. In KC patients, T_H_1 and T_H_2 cells may be produced in a positive feedback loop from naïve T cells and then inhibit each other; the immune response oscillates between T_H_1 and T_H_2 cells, triggering varying symptoms and eosinophil infiltration among chronic ketamine users.

Further, we found significantly increased levels of IL-6, TNF-*α*, and IFN-*γ* with comparably higher levels of IL-1*β*, IL-5, and IL-17 in the serum of KC patients than those of the healthy controls. Since the cytokine, IL-6, promotes the development of T_H_17 cells, the higher level of IL-6 in KC patients suggests that the balance between T_H_17 and T_H_1 responses as well as IL-6 production is dysregulated in KC, which resulted in the reinforced feedback that increases the IL-1*β* and IL-17 production in CD4+ T cells. This increase may contribute to disease pathogenesis (Figure 5). Therefore, the function of IL-6 might assist in the knowledge of its complex role in inflammation: throughout acute inflammation, the IL-6 might favor the resolution from neutrophilic infiltrate as well as favor the initiation from the immune response in chronic inflammation. Further, the IL-6 induces the development of T_H_17 cells from naïve T cells and, together with TGF- *β*, may boost the mononuclear-cell infiltrate and take part in disease pathogenesis [14].

T_REG_ cells have been recognized as a suppressor of effector T cells and, thus, are thought to control the T_H_1, T_H_2, and T_H_17 responses. Our results showed low concentration of T_REG_ related serum cytokines, IL-2 and TGF- *β*, which may explain the persistent inflammation in the KC patients (Figures 3(a) and 3(c)). It is to be noted that the TGF-*β*3 regulates molecules involved in cellular adhesion and extracellular matrix (ECM) formation in tissue [15]. So, the lower concentration of TGF-*β*3 in the KC group suggests the impaired healing capability of bladder epithelial lining in chronic KC patients. The elevated IL-17 level and reduced TGF-*β* level showed that the CD4+ T cell differentiation is biased away from T_REG_ cells toward the T_H_17 cell phenotype [16]. The differentiation of CD4^+^ cells generates the T_H_1 and T_H_2 phenotypes with the same receptor specificity, indicating the competition for antigenic stimulation, mediated by antigen-presenting cells (APCs), combined with cytokine-mediated cross-suppression between phenotypes to yield a response that is eventually dominated by T helper cells that are uniform in both receptor specificity and cytokine secretion phenotype. We found a different T helper cell phenotype distribution that the T_H_1, T_H_2, and T_H_17 receptors were all expressed in PBMCs (Figure 2), consistent with recent data [17]. We subsequently analyzed the serum cytokine secreted from the T helper cells in KC patients.

Previous studies have shown that many, but not all, of the effects of IFN-*γ* paracrine can be stimulated to T_H_1 cells to secrete lymphokines [18]. The increase in IFN-*γ* paracrine may stimulate the T_H_1 cells to secrete their own lymphokines TNF-*α*, IFN-*γ*, and IL-6 (Figure 3(b)). The IL-6, combined with TGF-*β* and IL-1*β*, induces the differentiation of T_H_17 from naïve cells and then enhances the IL-17 secretion (Figure 3(a)). IL-6 is an important switch that determines whether T_H_17 or T_REG_ cells are generated when TGF-*β* is present; TGF-*β*1 has been known to inhibit the differentiation of T_H_1 and T_H_2 cells [19, 20]. Hence, increases in IL-6 and IL-1*β* noted in KC patients may cause an offsetting response to the increase in TGF-*β*1, TGF-*β*2, and TGF-*β*3 (Figures 3(a) and 3(c)). IL-6 and IL-1*β* have been found to be increased in inflammatory urinary tract disease, indicating that IL-6 may also play an essential role in bladder dysfunction related to urinary tract infection [21].

IL-6 is a multifunctional cytokine which is produced at sites of tissue inflammation. It has been demonstrated that IL-6 derived from APCs is able to initiate IL-4 production in naïve CD4^+^ T cells, thereby polarizing these cells into T_H_2 cells [22]. IL-4 targets B cells and mast cells, resulting in the production of IgE and inhibits IL-6 production; IL-5 targets eosinophils. The increased IFN-*γ* may induce differentiation of CD4^+^ cells into T_H_1 cells and suppress the proliferation of T_H_2 cells, causing apoptosis of eosinophils and inhibiting IgE production [23]. Our results showed that both the eosinophil counts (Table 1) and IgE (Table 2) in KC patients were higher than that of normal laboratory values. Increased T_H_2 related cytokines, IL-4 and IL-5, were also found in KC patients, although there was no significant difference when compared to the normal control group (Figure 3(a)). The T_H_2 cells also inhibit the development and function of T_H_17 and T_H_1 cells by means of their signature cytokine IL-4 [22]. These results demonstrated that IL-6 signaling has the ability to enhance the production of T_H_2 related cytokines (i.e., IL-4 and IL-5) via CD4+ T cell pathway. As regards KC disease, a strong correlation between the frequencies of IL-17, TNF-*α*, and IFN-*γ* in KC patients has been observed (Figure 4). Previous studies have indicated that TNF-*α* and IL-17 have synergistic effects with many other factors, including IL-1*β*, IL-22, and IFN-*γ* [24]. The infiltrations of T_H_1 and T_H_17 cells have been associated with the pathogenesis of autoimmune disorders.

T_H_17 effector cells are induced in parallel to T_H_1, and, like T_H_1, polarized T_H_17 cells have the capacity to induce inflammation and autoimmune disease [16]. The main function of IL-17-secreting T cells is to mediate inflammation, by stimulating production of inflammatory cytokines that promote the recruitment of neutrophils and macrophages [25]. Although we observed elevated IL-1*β* levels, the reduction of TGF-*β* is consistent with the elevation of IL-6 and IL-17 and may reflect T_H_17 cell activity rather than T_REG_ cell activity. In addition, the elevated levels of T_H_2 cells and their associated effector cytokines, IL-5, were correlated with IL-1*β* and IL-17, indicating a central role for T_H_17-mediated immune responses associated with IL-6; IL-6 promotes T_H_17 but suppresses T_REG_ differentiation in KC disease [26]. Since the IL-5 and IL-17A regulate the production of specific-IgE* in vivo* [27], these results may explain the reason of increased IgE level and also can convince the eosinophil infiltration in the bladder suburothelium that was found in KC patients.

Study limitations include small sample size and the single time point serum measurement. The increased cytokine levels in KC patients reflect that the ongoing bladder inflammation noted at the bladder ulcer site is to be confirmed by further investigation. Further investigation is necessary to clarify the role of IL-6 in T_H_1/T_H_2/T_H_17-regulated signaling pathway in ketamine-induced cystitis. These preliminary results pieced together from data collected from ketamine patients offer a possible mechanism of ketamine-associated cystitis. However, further* in vivo* tests would confirm the effects of various cytokine concentrations on T cell differentiation, particularly the initial concentrations of TGF-*β* and IL-6.* In vivo* experiments would also allow us to control the daily amount of ketamine and the bladder could be analyzed at various stages of the inflammatory response to confirm our theory.

## 5. Conclusion

Ketamine may cause acute inflammation through T_H_1 pathway and elicit secondary mediator IL-6 generation which links to the dynamic balance of T_H_17/T_REG_ cell effector functions. The IL-6 may cause tissue destruction and collagen deposition via T_H_17 cells activation and suppress T_REG_ cells resulting in sustained activation of T_H_1 and T_H_2 cells. Further, the IL-6 may promote the differentiation of T_H_2 cells by enhancing endogenous IL-4 production and increase the production of IgE in KC patients. This is associated with T_H_2-mediated chronic inflammatory response. In addition, the low concentration of TGF-*β*3 in KC patients implied a suppression of T_REG_ function, which may cause the tissue damage and immune-mediated inflammation. This will subsequently impair the healing of urothelium in KC patients.

## Figures and Tables

**Table 1:** Demographic and laboratory characteristics of KC patients. ^*∗*^SD: standard deviation. The normal range of each characteristic is obtained from Department of Clinical Pathology, Tri-Service General Hospital, Taipei, Taiwan.

Characteristic	*n*, *n* (%) or mean (SD)^*∗*^	Unit	Normal range
Male : female ratio	31 : 22		—
Age, years	23.84 (4.74)		—
Duration of ketamine abuse, months	50.44 (4.99)		—
WBC	9.32 (0.45)	10 × 3/uL	4.50–11.00
Neutrophil	63.28 (1.5)	%	40.0–74.0
Lymphocyte	25.71 (1.15)	%	19.0–48.0
Monocyte	6.34 (0.37)	%	3.4–9.0
Eosinophil	4.11 (0.77)	%	0.0–7.0
pH	6.31 (0.1)	—	5.0–8.0
Urine RBC	6.87 (2.168)	HPF	0–5
Urine WBC	16.11 (4)	HPF	0–5
Epithelial cells	5.4 (1.91)	HPF	0–6
Creatinine	1.25 (0.42)	mg/dL	0.5–0.9

**Table 2:** Serum complement and immunoglobulin tests of KC patients. The normal range of each characteristic is obtained from Department of Clinical Pathology, Tri-Service General Hospital, Taipei, Taiwan.

Characteristic	*n*, *n* (%) or mean (SD)^*∗*^	Unit	Normal range
C3	96.48 (4.59)	mg/dL	79.0–152.0
C4	20.37 (1.01)	mg/dL	16.0–38.0
Immunoglobulin G	927.57 (51.48)	mg/dL	751–1560
Immunoglobulin M	108.86 (14.6)	mg/dL	46–304
Immunoglobulin E	261.59 (56.03)	IU/mL	<165.0

^*∗*^ *P* < 0.05.
